# Rational Design of Synergistic Structure Between Single-Atoms and Nanoparticles for CO_2_ Hydrogenation to Formate Under Ambient Conditions

**DOI:** 10.3389/fchem.2022.957412

**Published:** 2022-07-19

**Authors:** Shengliang Zhai, Ling Zhang, Jikai Sun, Lei Sun, Shuchao Jiang, Tie Yu, Dong Zhai, Chengcheng Liu, Zhen Li, Guoqing Ren

**Affiliations:** Institute of Molecular Sciences and Engineering, Institute of Frontier and Interdisciplinary Science, Shandong University, Qingdao, China

**Keywords:** synergistic effect, single-atom catalysts (SACs), CO_2_ hydrogenation, formic acid, ambient conditions

## Abstract

Single-atom catalysts (SACs) as the new frontier in heterogeneous catalysis have attracted increasing attention. However, the rational design of SACs with high catalytic activities for specified reactions still remains challenging. Herein, we report the rational design of a Pd_1_-Pd_NPs_ synergistic structure on *2,6*-pyridinedicarbonitrile-derived covalent triazine framework (*CTF*) as an efficient active site for CO_2_ hydrogenation to formate under ambient conditions. Compared with the catalysts mainly comprising Pd_1_ and Pd_NPs_, this hybrid catalyst presented significantly improved catalytic activity. By regulating the ratio of Pd_1_ to Pd_NPs_, we obtained the optimal catalytic activity with a formate formation rate of 3.66 mol_HCOOM_·mol_Pd_^−1^·h^−1^ under ambient conditions (30°C, 0.1 MPa). Moreover, as a heterogeneous catalyst, this hybrid catalyst is easily recovered and exhibits about a 20% decrease in the catalytic activity after five cycles. These findings are significant in elucidating new rational design principles for CO_2_ hydrogenation catalysts with superior activity and may open up the possibilities of converting CO_2_ under ambient conditions.

## Introduction

In recent years, single-atom catalysts (SACs) have attracted significant interest in heterogeneous catalysis for their advantages of 100% metal atom use, single active site structure, and unexpected high activity and selectivity for various reactions (Yang et al., 2013; Wang et al., 2018). Since the concept of SACs was proposed in 2011 (Qiao et al., 2011), it has solved the problems of low metal loadings (Liu et al., 2016; Li et al., 2018), poor thermal stability (Jones et al., 2016; Liu et al., 2020a; Liu et al., 2020b), and difficulties in large-scale production (Liu et al., 2020b; He et al., 2020) after nearly 10 years of vigorous development. However, although SACs are presented as homogeneous active centers, their catalytic activity is often difficult to compare with that of homogeneous catalysts and enzyme catalysts, and even lower than that of the corresponding nanocatalyst system in some cases (Ding et al., 2015). One of the reasons is that it is often difficult for a single metal atom center to activate multiple reactants with different properties or a single polyatomic molecule. The catalytic activity of monatomic catalysts does not depend on their single active center, but also on their surrounding chemical environment. The design and controllable construction of the synergistic structure between the single-atom center and its surrounding active sites is the key to achieving the high-efficiency catalytic performance of SACs.

Synergistic catalysis has been widely studied in the field of nanocatalyst and has shown obvious advantages in improving the activity and product selectivity of catalysts. For example, Liu et al. (2017) designed a Schiff-base-mediated gold catalyst for hydrogenation to formate reaction. The Schiff-base functional group grafted on a SiO_2_ support helps CO_2_ activation by the formation of a weak carbamate zwitterionic intermediate, and Au is for H_2_ dissociation. We previously introduced a CeO_2_ promoter with excellent dissociation ability to water molecules and oxygen molecules into an Au/MgGa_2_O_4_ catalyst to establish the synergistic catalytic effect between nano Au and the CeO_2_ promoter, which significantly improved the catalytic activity for the water gas shift and catalytic combustion reaction (Ren et al., 2019). As for SACs, although the concept of an atomic scaled synergistic effect has not been clearly proposed and systematically studied, there are some research examples of synergistic effects. For example, we recently reported a highly active dual single-Pd-atom catalyst, which could catalyze the hydrogenation of CO_2_ to formate under ambient conditions (Ren et al., 2022). It was found that the pore enrichment effect of microporous structures and the ternary synergetic effect among two neighboring Pd atoms and a rich nitrogen environment were the main reasons for this extraordinary catalytic activity. Liu et al. (2018) prepared a Ni-N-C SAC with metal loading up to 7.5 wt% by using N-doped C as the support, which exhibited excellent catalytic activity and cyclic stability for the hydrogenolysis of cellulose to ethylene glycol. The theoretical calculation results showed that the H_2_ molecule was activated by assistance of the nearest uncoordinated pyridine N atom of Ni. Therefore, the design of the synergistic catalytic structure based on the properties of reactants provides a good idea for the rational design of highly effective SACs.

In this work, we focused on the synergistic effect between single atoms and nanoparticles for CO_2_ hydrogenation to formate, an attractive reaction to achieve CO_2_ emission reduction and safe hydrogen storage (Alvarez et al., 2017; Eppinger and Huang, 2017; Su et al., 2019). Through theoretical calculations, we found that Pd_1_ and Pd_NPs_ were the preferred active sites for CO_2_ activation and hydrogen dissociation, respectively. Experimentally, we synthesized a Pd_1_-Pd_NPs_ synergistic structure on 2,6-pyridinedicarbonitrile-derived covalent triazine framework (*CTF*) by modulating the Pd loadings and reduction time, which exhibited high efficiency for the ambient hydrogenation of CO_2_ to formate. The optimal catalytic performance of the catalyst was obtained by regulating the ratio of Pd_1_ to Pd_NPs_. Furthermore, the recycling stability was also investigated. This work provides a new strategy for the rational design of highly active SACs and is very advantageous with regard to putting forward the conversion CO_2_ into practical applications.

## Experiment

### Chemicals

All the chemicals used in this study were of analytical grade and were used without further purification unless otherwise noted. Anhydrous zinc chloride (ZnCl_2_, ≥99%), 2,6-pyridinedicarbonitrile (2,6-DCP, ≥ 97%), and palladium trifluoroacetate (Pd(O_2_CCF_3_)_2_, ≥98%) were purchased from Shanghai Macklin Biochemical Co., Ltd. Hydrochloric acid (HCl, 36.0–38.0%) and sodium bicarbonate (NaHCO_3_, ≥99.5%) were purchased from Sinopharm Chemical Reagent Co., Ltd. High purity compressed N_2_, 10% H_2_/N_2_, and 50% CO_2_/50% H_2_ gases were obtained from the Deyi Gas Products Co., Ltd. (Qingdao, China).

### Sample Preparation

The *CTF-400:* 2,6-DCP-derived *CTF* was synthesized as described elsewhere (Kuhn et al., 2008). In detail, 2,6-pyridinedicarbonitrile and anhydrous ZnCl_2_ were mixed at the ratio of 1:5 (w/w) and ground under the glove box. The mixed powder was transferred into a quartz ampoule tube, and then evacuated, sealed, and heated to 400°C for 40 h. After the reaction, the mixture was subsequently ground and washed with large amounts of water and diluted HCl (2 M) to remove the residual ZnCl_2_. After that, the resulting black powder was dried in a vacuum at 150°C for 12 h, and the resulting product was denoted to be “*CTF-*400.”

*Pd/[CTF-400]:* Pd/[*CTF-400*] catalysts were prepared by soaking the *CTF-400* support powder in the aqueous solution of palladium trifluoroacetate with a Pd nominal weight loading of 10 wt% and 0.5 wt% for 12 h with magnetic stirring in a N_2_ atmosphere. The suspensions were then filtrated and washed with deionized water. The resulting filter cake was dried at 80°C for 12 h under a vacuum. The samples were denoted as *10*Pd/[*CTF-400*] and *0.5*Pd/[*CTF-400*], respectively, and the Pd actual weight loadings were detected to be 6.48 wt% and 0.28 wt% from ICP-AES analysis, respectively. *10*Pd/[*CTF-400*]*-*R*-t* samples were achieved by hydrogen reduction at 300°C for a different time. Specifically, the sample was heated to 300°C with the programming of 5°C/min under a constant flow of nitrogen. Then, the sample was kept at 300°C for 5, 10, 15, 30, 60, and 180 min under a constant flow of 10% H_2_/90% N_2_ at 10 ml/min, respectively. Subsequently, the furnace was cooled down to room temperature under the protection of nitrogen.

### Characterization

Fourier-transform infrared spectra (FT-IR) were performed in transmission mode on a Bruker VERTEX 70v spectrometer equipped with a DLATGS detector. The sample was diluted with KBr powder.

Surface areas and pore size distribution analyses were measured on Quantachrome Autosorb-iQ. N_2_ was used as the adsorbate and surface areas were calculated using the BET analysis method. All of the samples were degassed under a vacuum at 260°C for 8 h before measurement.

Elemental analyses were performed on a Vario El elemental analyzer.

X-ray photoelectron spectroscopy (XPS) data were analyzed on a Thermofisher ESCALAB 250Xi spectrometer using a monochromatized Al Kα X-ray source (1,486.6 eV). All the XPS data were calibrated by using C 1s binding energy at 284.8 eV.

Transmission electron microscopy (TEM) analysis was performed on FEI Tecnai G2 F20 at 200 keV. Aberration-corrected scanning transmission electron microscopy (AC-STEM) and EDX mapping analysis were performed on a JEOL JEM-ARM200F.

### Catalytic Reactions

CO_2_ hydrogenation to formate reaction was carried out in a base solution under ambient conditions (30°C and 1 bar). In general, 20 mg catalysts were added into 5 ml NaHCO_3_ (1 mol/L) in a three-necked bottle connected to a balloon. Then, the feed gas comprising CO_2_ (50% vol%) and H_2_ (50% vol%) was introduced after purging the residual air. After stirring for 12 h under ambient conditions, formate in the reaction mixture was determined by high-performance liquid chromatography (HPLC). In recycling experiments, the catalyst was recovered by filtration, washed with water, and dried under a vacuum. The reaction rate was calculated according to the following equation:

$$\text{Rate}=\;\frac{\text{Concentration of formate }\left(\frac{\text{mol}}{\text{L}}\right)\times \text{volume}\left(\text{L}\right)}{Pd\;amount\;\left(mol\right)\times time\left(h\right)}.$$

### Computational Methods

The calculation was performed by using the M06l/6-31G* method for nonmetal elements whereas the M06l/LANL2DZ method was used for metal atoms (Hehre et al., 1972; Hay and Wadt, 1985; Zhao and Truhlar, 2006). The metals were augmented with the corresponding LANL2DZ pseudo-potential, which was both acceptable in precision and time-consuming. Vibrational frequencies of the optimized configurations were analyzed to validate that these configurations correspond to the local minima or transition state (TS). The TS with one imaginary frequency was found and verified by the intrinsic reaction coordinate (IRC) method (Fukui, 1981).

The DFT calculations were performed on the Vienna ab initio simulation package (VASP) (Kresse and Furthmuller, 1996b; Kresse and Furthmuller, 1996a) to investigate the CO_2_ hydrogenation process on a Pd bulk surface and Pd_1_/CTF. The optB88-vdW was used to describe the exchange-correlation functional, which described the van der Waals forces appropriately (Dion et al., 2004; Lee et al., 2010). The projector augmented wave (PAW) potentials (Blochl, 1994) were used for electron–ion interactions, with a plane-wave kinetic energy cutoff of 400 eV. The geometry structures were relaxed until the forces on all atoms were less than 0.05 eV/Å. The transition states were searched using the Climbing Image Nudged Elastic Band (CI-NEB) method (Henkelman et al., 2000). Each transition state was relaxed until the forces on all atoms were less than 0.05 eV/Å.

The Pd (1 1 1) surface was modeled by a three-layer slab with a (4 × 4) surface unit cell and a vacuum thickness of 20 Å. The bottom two atomic layers of Pd (1 1 1) were fixed while the remaining layer, together with the adsorbates, were fully relaxed during relaxation. The lattice constant for Pd_1_/CTF is the same as for Pd, with a size of 13.79 Å × 13.79 Å × 24.51 Å. The Brillouin zone was sampled using a (2 × 2×1) k-point grid based on the Monkhorst-Pack (1976) scheme.

## Results and Discussion

### Theoretical Predictions

**FIGURE 1:**
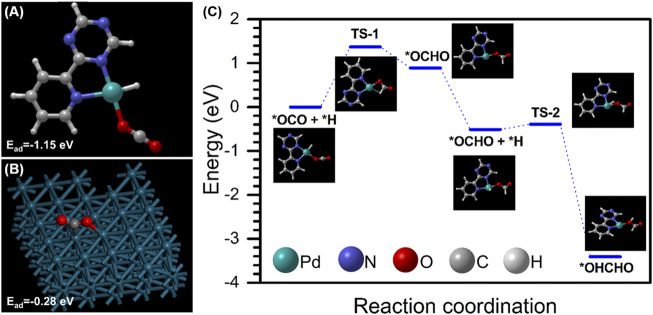
**(A,B)** CO_2_ adsorption energy on Pd_1_/*CTF* and the Pd surface; **(C)** the potential energy surface (PES) of CO_2_ hydrogenation on Pd_1_/*CTF*.

To achieve the most active catalyst structure, two different Pd species, Pd_1_ and Pd_NPs_, were investigated to carry out the CO_2_ activation and H_2_ dissociation process. We found that *2,6*-pyridinedicarbonitrile-derived *CTF-*coordinated Pd_1_ exhibited much higher adsorption energy for the CO_2_ molecule than that of the Pd (111) surface (−1.15 vs. −0.28 eV), indicating that Pd_1_ could be served as the active site for CO_2_ activation (Figures 1A,B). However, further calculation showed that the dissociation of the hydrogen molecule on Pd_1_ was difficult in thermodynamics. In contrast, the dissociation of the hydrogen molecule on the Pd surface is thermodynamically feasible (Supplementary Table S1). Moreover, metallic Pd has also been proved to have high activity in H_2_ dissociation with a nearly zero activation barrier (Ni and Zeng, 2009; Lozano et al., 2010). Therefore, we infer that Pd_1_ and Pd_NPs_ catalyze CO_2_ hydrogenation in collaboration through the hydrogen molecule dissociation on Pd_NPs_ and CO_2_ hydrogenation on Pd_1_. The potential energy surface (PES) of CO_2_ hydrogenation to formic acid on this hybrid catalyst is shown in Figure 1C. The first hydrogenation step is the rate-determining step with a barrier energy of 1.37 eV. The second step of hydrogenation is exothermic, with an energy of 2.90 eV. The energy barrier of this step is only 0.12 eV. The theoretical calculations confirm that the hydrogenation of CO_2_ catalyzed by the Pd_1_-Pd_NPs_ synergistic structure has a low barrier and can occur under ambient conditions.

### Catalyst Synthesis and Characterization

**FIGURE2:**
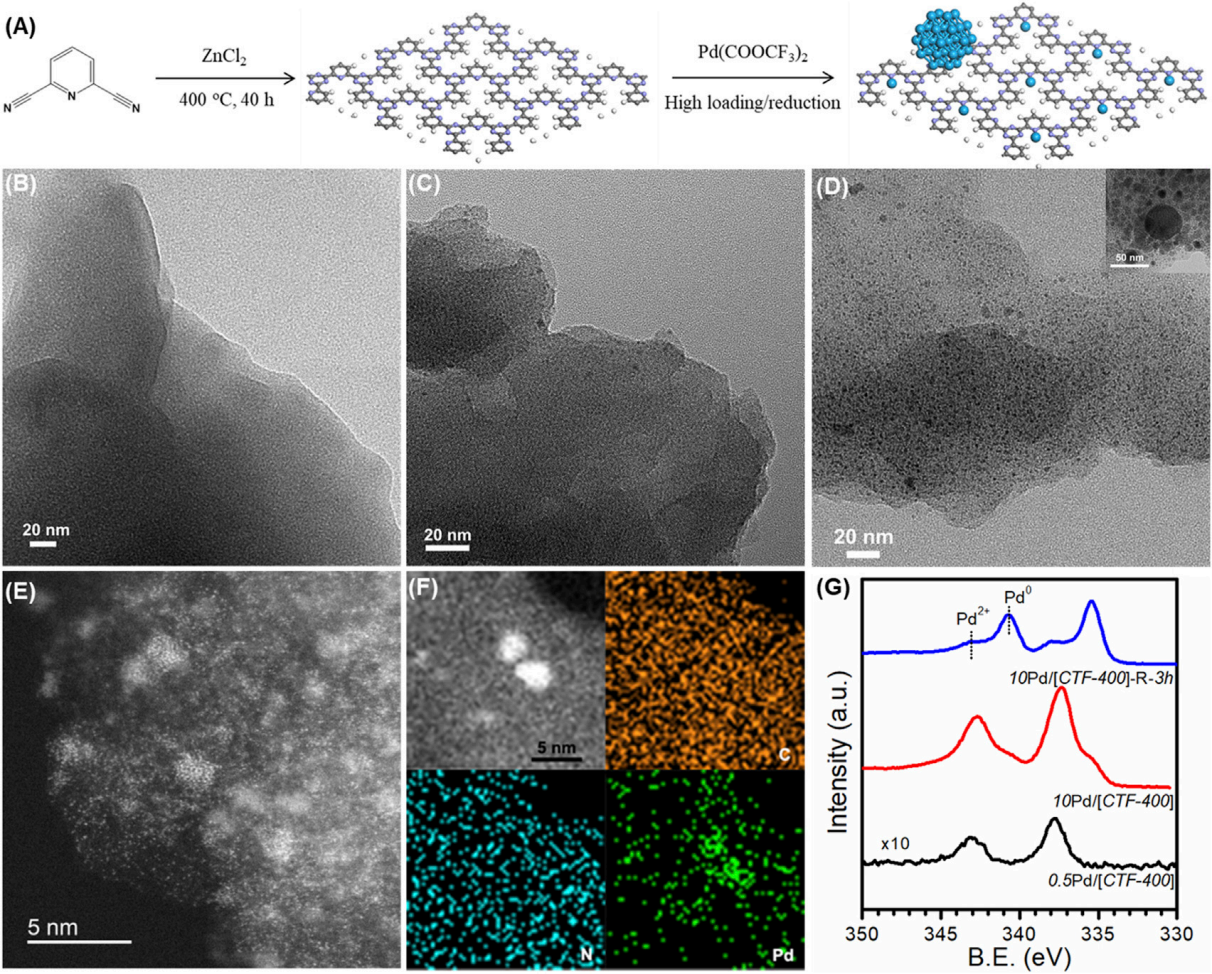
Structural characterizations of Pd/[*CTF-400*]. **(A)** Schematic illustration of the fabrication procedures of Pd/[*CTF-400*]. **(B–D)** HRTEM images of the characterization of *0.5*Pd/[*CTF-400*], *10*Pd/[*CTF-400*], and *10*Pd/[*CTF-400*]-R-*3h*. **(E,F)** Aberration-corrected STEM and EDS mapping of *10*Pd/[*CTF-400*]. **(G)** XPS profiles for *0.5*Pd/[*CTF-400*], *10*Pd/[*CTF-400*], and *10*Pd/[*CTF-400*]-R-*3h* samples.

The 2,6-pyridinedicarbonitrile-derived covalent triazine framework is formed by the trimerization of 2,6-pyridinedicarbonitrile in molten ZnCl_2_ at 400°C for 40 h, and is labeled *CTF-400* (Figure 2A). FT-IR, shown in Supplementary Figure S1, confirmed the formation of the corresponding covalent triazine rings. In addition, the prepared *CTF-400* possessed a surface area of 418 m^2^ g^−1^, a pore size of 0.53 nm, and a total pore volume of 0.21 m^3^ g^−1^ (Supplementary Table S2). The nitrogen content was estimated to be 19.51 wt% (Supplementary Table S3), which is high enough to load and stabilize the supported Pd species. The Pd/[*CTF-400*] catalyst was prepared by soaking the support powders in aqueous palladium trifluoroacetate followed by filtrating and washing after stirring for 12 h under a N_2_ atmosphere. By varying the initial Pd loading percent to be 0.5 wt% and 10 wt%, the single-atomic dispersed sample and the coexistence of Pd_1_ and Pd_NPs_ samples were prepared. The samples were denoted to be *0.5*Pd/[*CTF-400*] and *10*Pd/[*CTF-400*], respectively. After further reduction of *10*Pd/[*CTF-400*] at 300°C for 3 h, the Pd_NPs_-dominated sample was also achieved as the reference, which was denoted to be *10*Pd/[*CTF-400*]-R-*3h*.

Figures 2B–D display the TEM images at low magnification for *0.5*Pd/[*CTF-400*], *10*Pd/[*CTF-400*], and *10*Pd/[*CTF-400*]-R-*3h*, respectively. It is obvious that *0.5*Pd/[*CTF-400*] presents the notable character of *CTF-400* without any Pd nanoparticles, which indicates that Pd is atomically dispersed on *CTF-400* (Figure 2B). Scattered Pd nanoparticles appear on the *10*Pd/[*CTF-400*] sample, which illustrates that Pd atoms on *CTF-400* have partially aggregated to nanoparticles (Figure 2C). Further observation of this sample using AC-STEM confirms that Pd single atoms and nanoparticles coexist with good contact (Figures 2E,F). However, for the *10*Pd/[*CTF-400*]-R-*3h* sample, a large amount of Pd nanoparticles with a major size of ∼3 nm was presented. The formation of Pd_NPs_ was due to the aggregation of highly dispersed Pd atoms. For the as-prepared catalyst, most of the Pd species are well dispersed as single-atoms and sub-nanoparticles (as shown in Figures 2E,F), which have high surface energy and are unstable at high temperatures and reductive atmospheres. Therefore, the percentage of Pd_NPs_ in the *10*Pd/[*CTF-400*]-R-*3h* sample increased after reduction for 3 h. In addition, electronic properties for both samples were also estimated by XPS. As shown in Figure 2G, the binding energy of Pd 3*d*_*5/2*_ in *0.5*Pd/[*CTF-400*] is 337.3 eV, which can be attributed to that for Pd^2+^ presented as Pd_1_ in geometry. In addition to Pd^2+^, *10*Pd/[*CTF-400*] also shows the characteristic of metallic Pd corresponding to Pd_NPs_ in geometry with a binding energy of Pd 3*d*_*5/2*_ at 335.2 eV, and the ratio of Pd^2+^ to Pd^0^ is estimated to be about 90%. Different from that of *10*Pd/[*CTF-400*], Pd^0^ is dominant in the *10*Pd/[*CTF-400*]-R-*3h* sample with the ratio of Pd^0^ to Pd^2+^ being more than 70%. On the whole, the Pd_1_, Pd_1_-Pd_NPs_ hybrid, and Pd_NPs_-dominated catalysts were successfully prepared, which can be used as good model catalysts to study the synergistic effect between different Pd active sites.

### Catalytic Hydrogenation of CO_2_ to Formic Acid Under Ambient Conditions

**FIGURE 3:**
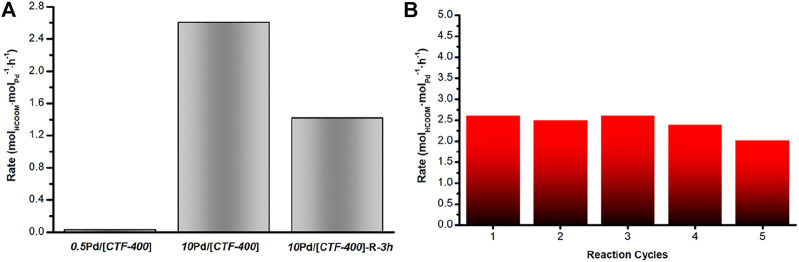
Catalytic performance of Pd/[*CTF-400*] for the hydrogenation of carbon dioxide to formate. **(A)** Formate formation rate of *0.5*Pd/[*CTF-400*], *10*Pd/[*CTF-400*], and *10*Pd/[*CTF-400*]-R-*3h*. **(B)** Reuse test of *10*Pd/[*CTF-400*]. Reaction conditions: 20 mg catalyst, 30°C, 1bar (CO_2_/H_2_ = 1:1), and 5 ml NaHCO_3_ (1 M), 12 h.

The catalytic performances of the as-prepared Pd catalysts for CO_2_ hydrogenation were studied at 30°C in a H_2_/CO_2_ mixture (0.1 MPa) with NaHCO_3_ (1 mol/L) as an additive in the liquid phase. After a reaction of 12 h, the formate was detected by using HPLC. As shown in Figure 3A and Table 1, compared with the Pd_1_ and Pd_NPs_ nanoparticle-dominated catalysts, the CO_2_ hydrogenation activity of the *10*Pd/[*CTF-400*] catalyst was significantly enhanced (entries 1, 2 and 8 in Table 1). In detail, *10*Pd/[*CTF-400*] exhibited a formate formation rate of 2.60 mol_HCOOM_·mol_Pd_^−1^·h^−1^, which was obviously higher than that of *0.5*Pd/[*CTF-400*] (0.026 mol_HCOOM_·mol_Pd_^−1^·h^−1^) and *10*Pd/[*CTF-400*]-R-*3h* (1.42 mol_HCOOM_·mol_Pd_^−1^·h^−1^). Moreover, the heterogeneous nature of this catalyst allows it to be easily recovered by centrifugation, and the recycling tests indicated that there was around a 20% decrease in the catalytic activity after five uses (Figure 3B). This result demonstrates that the catalyst can be reused after a simple separation process, which is very advantageous with regard to practical applications.

**TABLE 1:** Performance of Pd/[*CTF-400*] for CO_2_ hydrogenation to formate.

Entry	Catalyst	HCOOH/mM	Rate (mol_HCOOM_·mol_Pd_^−1^·h^−1^)
1	0.5Pd/[CTF-400]	0.033	0.026
2	10Pd/[CTF-400]	76.4	2.60
3	10Pd/[CTF-400]-R-5 min	86.3	2.86
4	10Pd/[CTF-400]-R-10 min	110.3	3.66
5	10Pd/[CTF-400]-R-15 min	102.2	3.39
6	10Pd/[CTF-400]-R-30 min	88.9	2.94
7	10Pd/[CTF-400]-R-1 h	58.7	1.95
8	10Pd/[CTF-400]-R-3 h	42.8	1.42

Reaction condition: 20 mg catalyst, 5 ml 1M NaHCO_3_ solvent, 30°C, 0.1 MPa, 12 h.

**FIGURE 4:**
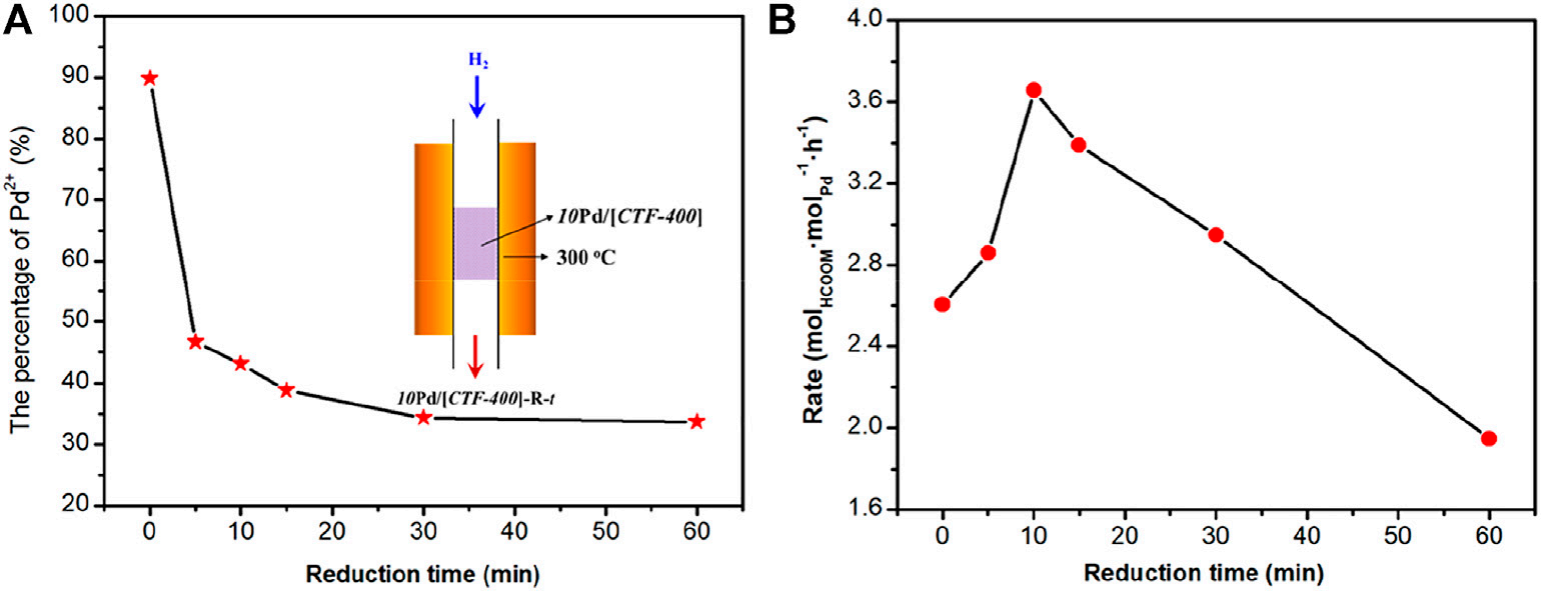
Optimization of the ratio of Pd single-atoms to nanoparticles for the hydrogenation of carbon dioxide to formate. **(A)** Percentage of Pd_1_ in *10*Pd/[*CTF-400*]-R-*t* samples. **(B)** Formate formation rate of *10*Pd/[*CTF-400*]-R-*t* samples (*t* = 0, 5, 10, 15, 30, and 60 min).

The aforementioned result confirms the theoretical predictions that the coexistence of Pd_1_ and Pd_NPs_ is necessary for the activation of H_2_ and CO_2_ at the same time. To obtain the optimized catalytic activity, the ratio of Pd_1_ to Pd_NPs_ was optimized. We attempted to get the sample with optimal Pd_1_/Pd_NPs_ by lowering the *10*Pd/[*CTF-400*] sample into a tubular furnace at 300°C and reducing it in 10 vol% H_2_ for 5, 10, 15, 30, and 60 min, respectively. The corresponding samples are denoted as *10*Pd/[*CTF-400*]-R-*t*, where “*t*” represents the reduction time. X-ray photoelectron spectroscopy (XPS) was performed to get the ratios between Pd single atoms and nanoparticles (Figure 4A and Supplementary Figures S3–S7). With careful deconvolution from the overlapped peaks of Pd^2+^ 3d and Pd^0^ 3d, the ratios of Pd_1_ ion to the total palladium were estimated to be 89.9, 46.7, 43.1, 38.7, 34.3, and 33.7% with the continuous extension of hydrogen reduction time from 0 to 60 min (Figure 4A). Figure 4B shows the catalytic performances of *10*Pd/[*CTF-400*]-R-*t* under ambient conditions (30°C, 0.1 MPa). It can be seen that the catalytic activities exhibit a volcano-type curve with a decrease in the Pd_1_ ratio. *10*Pd/[*CTF-400*]-R-*10* min with a Pd_1_ ratio of 43.1% performs the best catalytic activity, and the formate formation rate reaches 3.66 mol_HCOOM_·mol_Pd_^−1^·h^−1^. The existence of the optimal Pd_1_ ratio is due to the rate equilibrium of hydrogen dissociation on Pd^0^ nanoparticles and carbon dioxide activation on Pd_1_.

**FIGURE 5:**
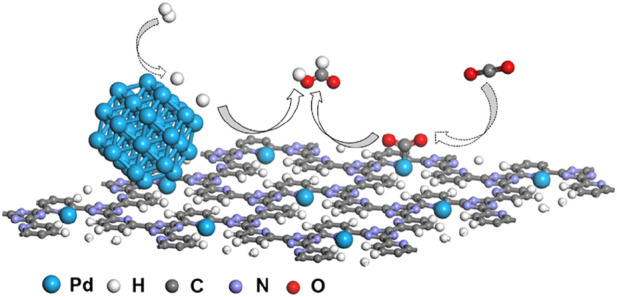
Proposed synergistic effect mechanism of Pd/[*CTF-400*] for the hydrogenation of carbon dioxide to formate.

Based on the aforementioned analysis, the synergistic effect mechanism of *10*Pd/[*CTF-400*] for the hydrogenation of CO_2_ to formate is shown in Figure 5. The *10*Pd/[*CTF-400*] catalyst integrates both Pd_NPs_ and Pd_1_ into one catalyst system, where the Pd_NPs_ boosts the dissociation of H_2_ whereas Pd_1_ ions undertake the activation task of CO_2_. Through the atom diffusion process, H atoms generated at Pd_NPs_ move to the adsorbed CO_2_ on Pd_1_ for high-efficiency hydrogenation. Theoretical calculations shown in Figure 1 confirmed the rationality of the tasks over each active site, that is, hydrogen dissociation occurs more easily on Pd_NP_, and carbon dioxide hydrogenation activation prefers to occur on Pd_1_. Experiments further verified the synergistic effect between Pd_1_ and Pd_NPs_, and the 10Pd/[*CTF-400*] catalyst performed nearly two orders of magnitude higher activity than *0.5*Pd/[*CTF-400*] and twice the reactivity of 10Pd/[*CTF-400*]-R-3 h.

## Conclusion

In summary, we have rationally designed a highly efficient catalytic system for the hydrogenation of carbon dioxide to formate under ambient conditions based on theoretical predictions. Through modeling the CO_2_ adsorption and hydrogen dissociation process on both Pd_1_ and Pd_NPs_, it was found that Pd_1_ performed the higher adsorption energy for CO_2_ and could be a potential candidate for CO_2_ activation. Compared with Pd_1_, hydrogen dissociation occurred more easily on Pd nanoparticles. Based on this prediction, the Pd/[*CTF-400*] catalyst integrating both Pd_1_ and Pd_NPs_ on one catalyst system was synthesized and realized the hydrogenation of CO_2_ to formate with a formate formation rate of 3.66 mol_HCOOM_·mol_Pd_^−1^·h^−1^ under ambient conditions (30°C, 1 bar). This hybrid catalyst presented nearly two orders of magnitude higher than the catalyst containing bare Pd_1_ and twice the reactivity of that containing bare Pd^0^ nanoparticles. These discoveries may pave the way for the construction of active SACs with synergistic effects and open up the possibilities of converting CO_2_ under ambient conditions.

## Data Availability

The original contributions presented in the study are included in the article/Supplementary Material; further inquiries can be directed to the corresponding author.
