# Supplementary material for: Rational Design of Synergistic Structure Between Single-Atoms and Nanoparticles for CO2 Hydrogenation to Formate Under Ambient Conditions
Source: Front Chem. 2022 Jul 19;10:957412. doi: 10.3389/fchem.2022.957412 (PMC9343707; doi:10.3389/fchem.2022.957412)
Supplement: Supplementary file 1 [file DataSheet1.DOCX]

Supplementary Material

Rational Design of Synergistic Structure between Single-atoms and Nanoparticles for CO_2_ Hydrogenation to Formate under Ambient Conditions

Shengliang Zhai ^1, #^, Ling Zhang ^1, #^, Jikai Sun ^1, #^, Lei Sun ^1^, Shuchao Jiang ^1^, Tie Yu ^1, *^, Dong Zhai ^1^, Chengcheng Liu ^1^, Zhen Li ^1^, Guoqing Ren ^1, *^

^1^ *Institute of Molecular Sciences and Engineering, Institute of Frontier and Interdisciplinary Science, Shandong University, Qingdao, Shandong, 266237, P. R. China*

^#^ These authors contributed equally to this work.

^*^ Corresponding author

*Tie Yu:* yutie@sdu.edu.cn, *Guoqing Ren:* renguoqing@sdu.edu.cn





**Supplementary Figure S1|** FT-IR spectra of the monomer *2,6-DCP* and *CTF-400* samples


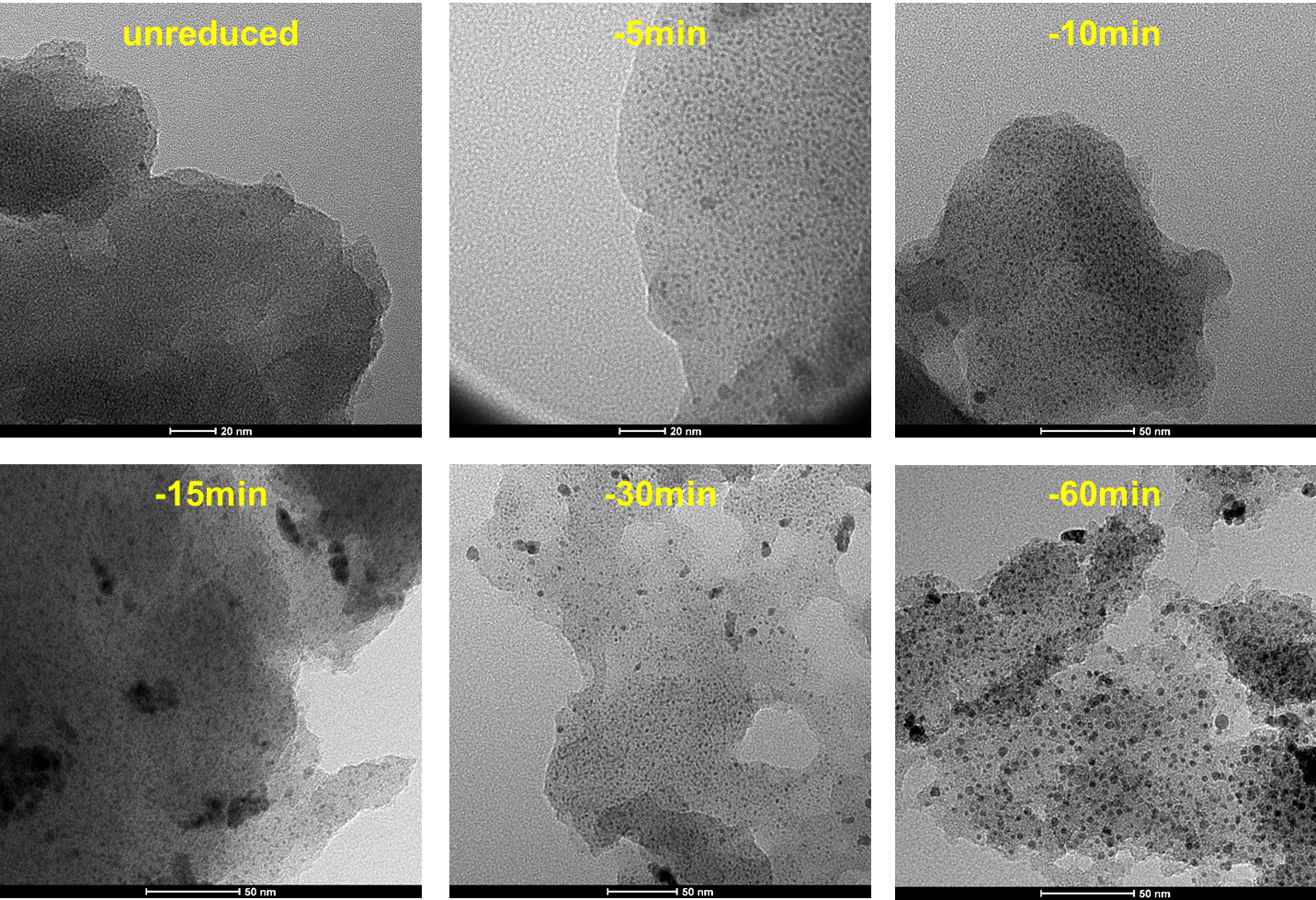


**Supplementary Figure S2|** HRTEM images of *10*Pd/[*CTF-400*]-R-*t*





**Supplementary Figure S3|** X-ray photoelectron spectrum of Pd 3d for*10*/[*CTF-400*]-R-*5min*.





**Supplementary Figure S4|** X-ray photoelectron spectrum of Pd 3d for *10*Pd/[*CTF-400*]-R-*10min*.





**Supplementary Figure S5|** X-ray photoelectron spectrum of Pd 3d for *10*Pd/[*CTF-400*]-R-*15min*.





**Supplementary Figure S6|** X-ray photoelectron spectrum of Pd 3d for *10*Pd/[*CTF-400*]-R-*30min*.





**Supplementary Figure S7|** X-ray photoelectron spectrum of Pd 3d for *10*Pd/[*CTF-400*]-R-*1h*.

**Supplementary Table S1|** H_2_ dissociation energy on Pd surface and Pd_1_/CTF

| Materials | E_ad_ (eV) | E_dis_ (eV) |
| --- | --- | --- |
| Pd_1_/CTF | -0.89 | / ^a^ |
| Pd-surface | -0.09 | 0.77 |

^a^ Hydrogen dissociation cannot occur on *2,6-DCP-CTF*-[Pd] thermodynamics.

**Supplementary Table S2|** Porosity data for *CTF-400* from N_2_ isotherms at 77 K

| Materials | Surface area^‡^  (m^2^·g^-1^) | Total pore volume ^¶^  (m^3^·g^-1^) | Pore size^§^  (nm) |
| --- | --- | --- | --- |
| *CTF-400* | 418 | 0.21 | 0.53 |
|  |  |  |  |

^‡^ Determined by the BET method.

^¶^ Determined for *P*/*P*_0_=0.99.

^§^ Determined by using SF model.

**Supplementary Table S3|** Elemental analysis of *CTF-400*

| Materials | C (wt.%) | N (wt.%) | H (wt.%) |
| --- | --- | --- | --- |
| *CTF-400* | 45.62 | 19.51 | 3.82 |
|  |  |  |  |
